# Complete genome sequence of the *Klebsiella oxytoca* phage vB_KoxiM_BaqKoxi isolated from the Magdalena River, Atlántico, Colombia

**DOI:** 10.1128/mra.00947-25

**Published:** 2026-02-05

**Authors:** Dayan Lozano-Solano, Jhonatan Reales-González, Leydis Vides-Castro, Antonio J. Acosta-Hoyos

**Affiliations:** 1 Universidad Simón Bolívar, Centro de Investigaciones en Ciencias de la Vida - CICV, Barranquilla, Colombia; 2 School of Chemistry and Molecular Bioscience and Molecular Horizons, University of Wollongong 8691, Wollongong, NSW, Australia; 3 Estudiante de Maestría en Genética, Facultad de Ciencias Básicas y Biomédicas, Universidad Simón Bolívar 125419, Barranquilla, Colombia; University of Wisconsin-Madison, Madison, Wisconsin, USA

**Keywords:** bacteriophages, depolymerase, *Klebsiella oxytoca*

## Abstract

We report the genome of vB_KoxiM_BaqKoxi (BaqKoxi), a temperate phage infecting an antibiotic-resistant strain of *Klebsiella oxytoca,* isolated from the Magdalena River in Barranquilla, Colombia. BaqKoxi contains a linear 72,063 bp dsDNA genome with 98 coding DNA sequences (CDS), including two depolymerases with complementary activities, a pectate lyase, and a capsular depolymerase.

## ANNOUNCEMENT

*Klebsiella oxytoca* is considered an opportunistic human pathogen that can cause various infections, including hemorrhagic colitis, urinary tract infections, and antibiotic-associated infections (1). Consequently, the World Health Organization (WHO) included Klebsiella in the Bacterial Priority Pathogens List (2). Phage therapy emerged as a promising strategy to combat bacterial infections (3, 4).

BaqKoxi was obtained using a clinical strain of *Klebsiella oxytoca*, which was isolated from a urinary infection sample and stored in the Caribbean Biobank of Universidad Simón Bolívar in Barranquilla, Colombia, under the name 013. We collected 10 mL of water from the Magdalena River in Barranquilla, Colombia (10.9639°N, 74.7964°W) on 18 September 2019, filtered through a 0.22-μm membrane, and mixed with 10 mL of lysogeny broth (LB) and 100 µL of an overnight culture of 013, and incubated at 37°C with shaking at 180 RPM for 24 h. The phage plaques were propagated in LB medium supplemented with 0.05 mM CaCl_2_ using the double-layer overlay technique (5).

Genomic DNA was extracted from 100 µL of filtered phage lysate obtained after propagation in a bacterial host culture, followed by clarification and filtration to remove residual cellular debris. DNA was purified using the PureLink Viral RNA/DNA Mini Kit (Thermo Fisher Scientific) according to the manufacturer’s instructions. Whole-genome sequencing followed a previously described workflow (6). Briefly, DNA was fragmented by ultrasonication to ~550 bp, and a library was prepared using the NEXTflex Rapid DNA Sequencing Kit. Fragments between 500 and 750 bp were selected using a BluePippin system (Sage Science), pooled with PhiX control DNA, and sequenced on an Illumina MiSeq platform using a v3 flow cell. DNA quantity and library integrity were assessed using a Qubit fluorometer and an Agilent 2100 Bioanalyzer.

A total of 2,358,333 paired-end raw reads were generated. The adapters and low-quality sequences (<Q30) were identified with FastQC (UseGalaxy.eu) and removed with Trimmomatic, along with reads <50 bp. Filtered reads were assembled *de novo* using SPAdes v3.12.0. Assembly yielded a 72,063 bp contig with the highest k-mer coverage (7.6×), consistent with the complete phage genome. Annotation with RAST-Tk v.1.073 (7) for the identification of the coding DNA sequences (CDS) and predicted putative functions. Default parameters were used unless otherwise noted.

**Fig 1:**
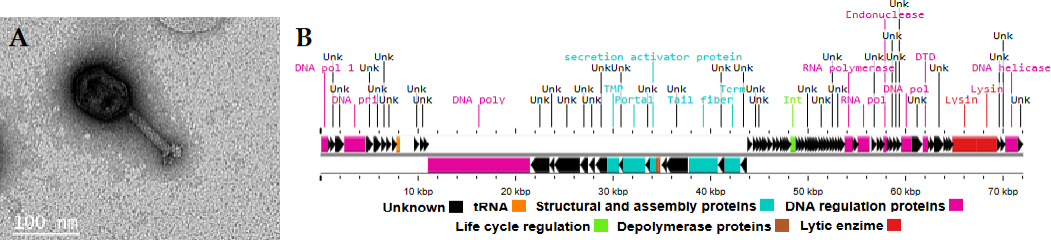
Virion morphology of vB_KoxiM_BaqKoxi. (**A**) Virion morphology and (**B**) genome modular structure. The virion morphology was determined through electron microscopy. A high-titer lysate was applied to Formvar-coated grids, negatively stained with 2% uranyl acetate at an accelerating voltage of 200 kV, and 195 k× magnification was used. The images were captured with a Zeiss EM-109 transmission electron microscope (Carl Zeiss AG), corresponding to a Myovirus. The genomic map was generated using Proksee (11) based on the BaqKoxi annotated genome.

BaqKoxi displays a Myovirus morphotype (Fig. 1A). It has a double-stranded DNA linear genome with short direct terminal repeats like T7 phage, determined through PhageTerm2 (8), and has a genome of 72,063 bp, with a GC content of 44.1%, and 98 CDS, of which 24 had a predicted function. The genome is organized into functional modules based on RAST-Tk (Fig. 1B). These include modules: structural and assembly proteins (8 CDS); DNA regulation proteins (13 CDS); life cycle regulation (1 CDS); lytic enzyme (2 CDS), and exhibit two depolymerase proteins codified by CDS 30 and 31, which have pectate lyase and a capsular depolymerase function, respectively (9, 10).

## Data Availability

The genome sequence of BaqKoxi has been deposited in GenBank under accession number PQ741797. The raw sequence reads have been submitted to the NCBI SRA under accession number PRJNA1288435.
